# Emergency of Primary NNRTI Resistance Mutations without Antiretroviral Selective Pressure in a HAART-Treated Child

**DOI:** 10.1371/journal.pone.0004806

**Published:** 2009-03-11

**Authors:** Elizabeth S. Machado, Adriana O. Afonso, Dwight V. Nissley, Philippe Lemey, Silvia M. Cunha, Ricardo H. Oliveira, Marcelo A. Soares

**Affiliations:** 1 Hospital Universitário Clementino Fraga Filho, Universidade Federal do Rio de Janeiro, Rio de Janeiro, Brazil; 2 Departamento de Genética, Universidade Federal do Rio de Janeiro, Rio de Janeiro, Brazil; 3 Gene Regulation and Chromosome Biology Laboratory, NCI-Fredrick, Frederick, Maryland, United States of America; 4 Basic Science Program, SAIC-Frederick, Inc., NCI-Fredrick, Frederick, Maryland, United States of America; 5 Department of Zoology, Oxford University, Oxford, United Kingdom; 6 Instituto de Puericultura e Pediatria Martagão Gesteira, Universidade Federal do Rio de Janeiro, Rio de Janeiro, Brazil; 7 Divisão de Genética, Instituto Nacional de Câncer, Rio de Janeiro, Brazil; University of Sao Paulo, Brazil

## Abstract

**Objective:**

The use of antiretrovirals (ARV) during pregnancy has drastically reduced the rate of the human immunodeficiency virus perinatal transmission (MTCT). As a consequence of widespread ARV use, transmission of drug resistant strains from mothers to their babies is increasing. Ultra-sensitive PCR techniques have permitted the quantification of minority viral populations, but little is known about the transmission of drug-resistant HIV-1 minority population in the setting of MTCT.

**Methodology/Principal Findings:**

We describe the case of a female child born to an HIV-infected mother, which had not taken any ARV during the pregnancy. The child's first genotype demonstrated a minor non-nucleoside reverse transcriptase inhibitor (K101E), and during her treatment with reverse transcriptase and protease inhibitors full resistance to non-nucleoside reverse transcriptase inhibitors (NNRTI) emerged (G190A). Phenotypic/genotypic analysis of variant quasispecies through yeast TyHRT assay was conducted to characterize minority resistant viral strains circulating in both mother and child. Maximum likelihood and Bayesian MCMC phylogenetic analyses were performed with samples from the pair to assess genetic relatedness among minor viral strains. The analysis showed that the child received a minor NNRTI resistant variant, containing the mutation K101E that was present in less than 1% of the mother's quasispecies. Phylogenetic analyses have suggested common ancestry between the mother's virus strain carrying K101E with the viral sequences from the child.

**Conclusion:**

This is the first documentation of MTCT of a minority resistant strain of HIV-1. The transmission of minor resistant variants carries the threat of emergence of multi-drug primary mutations without identified specific selective pressures.

## Introduction

The use of antiretrovirals (ARV), particularly the combination therapy known as highly active antiretroviral therapy (HAART) during pregnancy, has substantially decreased mother-to-child transmission (MTCT). In addition to AZT prophylaxis during delivery and to the newborn, C-section when viral suppression is not achieved and avoidance of breastfeeding has reduced the rate of MTCT to less than 2% [1]. As a consequence of ARV selective pressure and widespread use, transmission of resistance strains from mothers to their babies is increasing [2].

Vertical transmission of HIV-1 variants resistant to reverse transcriptase inhibitors has been reported and some studies suggest that resistant mutations are selectively transmitted [3]–[5]. More recently, however, no vertical transmission of NNRTI and PI major mutations has been observed, even when representing the predominant maternal variant [5]. Studies using single-dose nevirapine to MTCT prevention suggest that transmission of NNRTI-resistant strains is a rare event, if it occurs at all [6]–[7]. Transmission of major NNRTI and PI mutations has been rarely reported in infants born to mothers who acquired primary resistance mutations by heterosexual transmission or during the course of their treatment [8]–[9]. Transmitted resistant mutations can take years to fade away even when the population transmitted is a mixture of wild-type and drug-resistant virus [10].

Little is known about the transmission of drug-resistant HIV-1 minority population in the setting of MTCT. We report here a possible transmission of a minor variant carrying an NNRTI resistance mutation from an ARV-naïve mother to a child and subsequent emergence of this variant as a dominant population during an NNRTI non-based ARV treatment.

## Methods

### Case description

P50 is a female child born on May 1, 1999 and diagnosed with HIV on January 29, 2002. She was the index case of HIV-1 in the family. Her parents had not taken any ARV at the time of the child's diagnosis and her mother did not take any ARV during the pregnancy. The child was not breastfed. Her first CD4^+^ T-cell count was 308/µL (14%) and her viral load (VL) was 390,000 copies/ml. She was started on zidovudine and didanosine in August, 2002. One month after initiation of treatment her CD4^+^ counts rose to 28% and her VL was 1,700 HIV-RNA copies/ml, but DDI was changed for lamivudine and nelfinavir due to intolerance. She experienced immunological improvement during HAART although her VL was never undetectable. Her baseline genotyping test before ARV therapy showed the K101E RT mutation. A second genotyping performed one month after HAART showed K101E, G190A, and T215F. Another test performed 13 months after HAART initiation revealed the persistence of G190A and K101E and the accumulation of various NRTI mutations (Table 1).

**Table 1:** Evolution of resistance mutation profile of child P50, detected by standard genotyping.

Age	Treatment History	Standard Genotyping*
2 yrs 8 months	HIV diagnosis	
3 yrs	No ARV	K101E
3 yrs 3 months	AZT+DDI	
3 years 4 months	AZT+3TC+NFV	
3 yrs 5 months	AZT+3TC+NFV	K101E, G190A, T215F
4 years 5 months	AZT+3TC+NFV	M41L, D67N, T69N, K70R, K101E, M184V, G190A, T215F , K219E

* Only major drug resistance mutations are listed.

ARV = antiretrovirals, AZT = zidovudine, DDI = didanosine, 3TC = lamivudine, NFV = nelfinavir.

In order to evaluate whether the NNRTI primary mutation, G190A, was already presented in the child's quasispecies before the introduction of ARV, we analyzed the child's sample before the initiation of therapy. One sample of the mother (M50), collected on October, 2005, while she was still ARV-naïve was also analyzed for minor variants. Written informed consent was obtained from the mother prior to inclusion of both mother and child in the study.

### Phenotypic/Genotypic analysis of quasispecies variants through yeast TyHRT assay

Patient-derived HIV-1 RT domain DNA encompassing codons 37–250 was isolated through nested PCR from infected PBMC genomic DNA using primers Dp10 (5′CAACTCCCTCTCAGAAGCAGGAGCCG3′) and PolM4 (5′TATGTAGATGGGGC AGCTAACAG3′) for the first round, and primers Dp16 (5′CCTCAAATCACTCTTTGG CAAC3′) and RT-20 (5′GAAGAAGCAGAGCTAGAACTGGCAG3′) for the second round. RT sequences were incorporated into TyHRT elements by *in vivo* homologous recombination following transformation into *Saccharomyces cervisiae*. HIV-1 RT assays using TyHRT elements in yeast [11] and the construction of RT domain libraries [12] were carried out as described previously. Clones were assayed in the absence and presence of efavirenz and nevirapine (obtained from the NIH AIDS Research and Reference Reagent Program) to monitor RT activity and NNRTI susceptibility. Nevirapine- and efavirenz-resistant RTs with increased activity in the presence of inhibitor were characterized further.

HIV-1 RT domains of interest were isolated and sequenced as described previously [13]. RTs were amplified using primers A-35 (5′GAACCTCCGAGATCGAAGA3′) and I1097 (5′GCACTGCCTCTGTTAATTGT3′) and sequenced with internal primers 44F (5′GGATGGATGGCCCAAAAGT3′) and J801 (5′ATCCCTGGGTAAATCTGACT3′).

### Phylogenetic analyses

Phylogenetic analysis was performed using both maximum likelihood (ML) and Bayesian inference algorithms. To establish the epidemiological linkage of the M50-P50 mother-child pair, additional subtype B control sequences were obtained from the same geographical region (14 sequences form HIV infected children in Rio de Janeiro) and using BLAST similarity searches in GenBank (49 sequences). Analyses for the M50-P50 sequences were performed with and without codon 101 to avoid the effect of convergent evolution of the K101E substitution. ML trees were inferred using PhyML [14], employing a General Time Reversible model with discrete gamma-distributed rate variation among sites (GTR-γ; 4 categories). Bootstrap analysis was performed using 500 replicates. Bayesian phylogenetic analysis was performed using MrBayes with the same model settings as the ML analyses. Two independent Markov Chain Monte Carlo (MCMC) runs were carried out, each consisting of 4 chains and a default heating scheme. ML trees and MCMC consensus trees were visualized in FigTree (http://tree.bio.ed.ac.uk/software/figtree/).

*Ethics Statement*

## Results

### HIV-1 Quasispecies analyses

A total of 680 isolates with active RT from the child (referred as P50) and 2,931 sequences from the mother (M50) were tested. With respect to resistance phenotype, P50 showed 46 isolates associated with resistance to nevirapine and twenty resistant to efavirenz. Among M50 sequences, 22 were associated with resistance to nevirapine and 16 to efavirenz. None of 344 NNRTI sensitive clones from M50 sequenced showed the K101E mutation. Successful sequencing of resistant clones was obtained for 14 NNRTI-resistant variants from P50 and 30 from M50.

Among P50 sequences the mutations A98S, K101E, I135T and E138A were observed in all isolates. In seven of these sequences the G190A mutation was present. Other polymorphisms (L100I, L100S, V106A) were observed in individual sequences but were not associated with G190A mutation. M50 sequences also showed mutations A98S, I135T and E138A. NNRTI-resistant V106A and K103N variants were seen in two sequences, Y181C and Y188C in two sequences, and Y188H in five sequences. K101E was found in one sequence.

### Phylogenetic relatedness between M50 and P50 HIV-1 sequences

**Figure 1:**
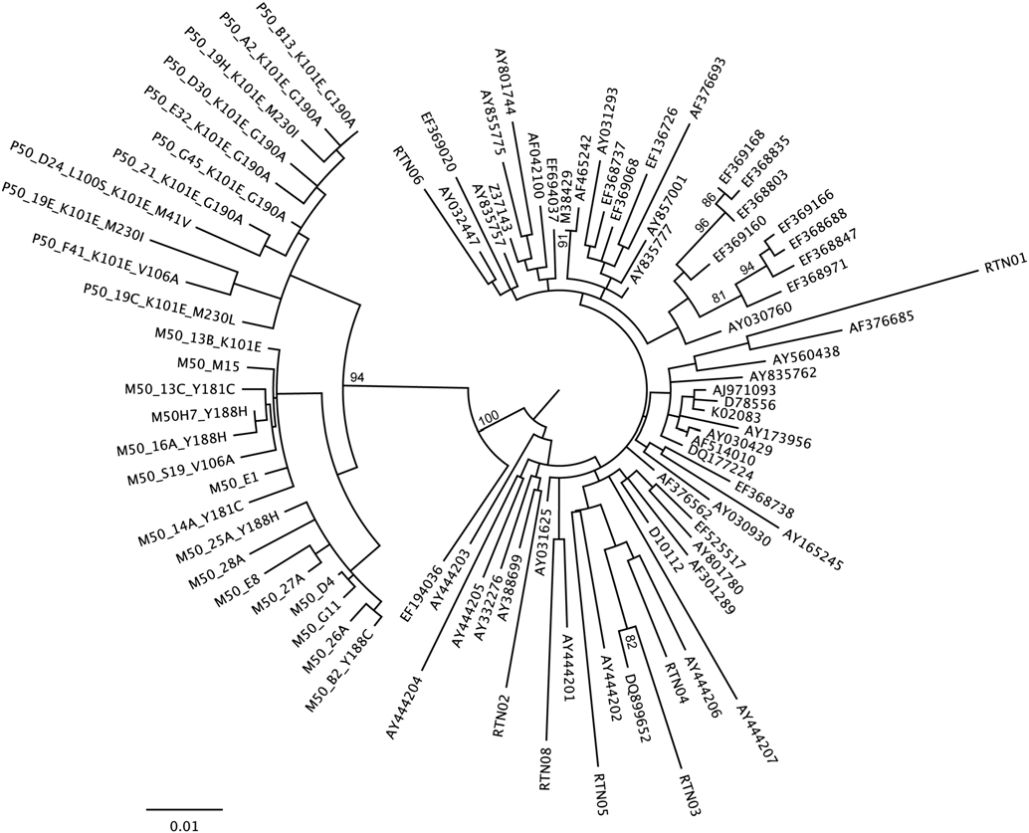
Maximum likelihood tree with bootstrap support values for the M50-P50 transmission chain including control sequences. Only bootstrap support values over 70% are shown. The Bayesian phylogenetic inference produced very similar results.

**Figure 2:**
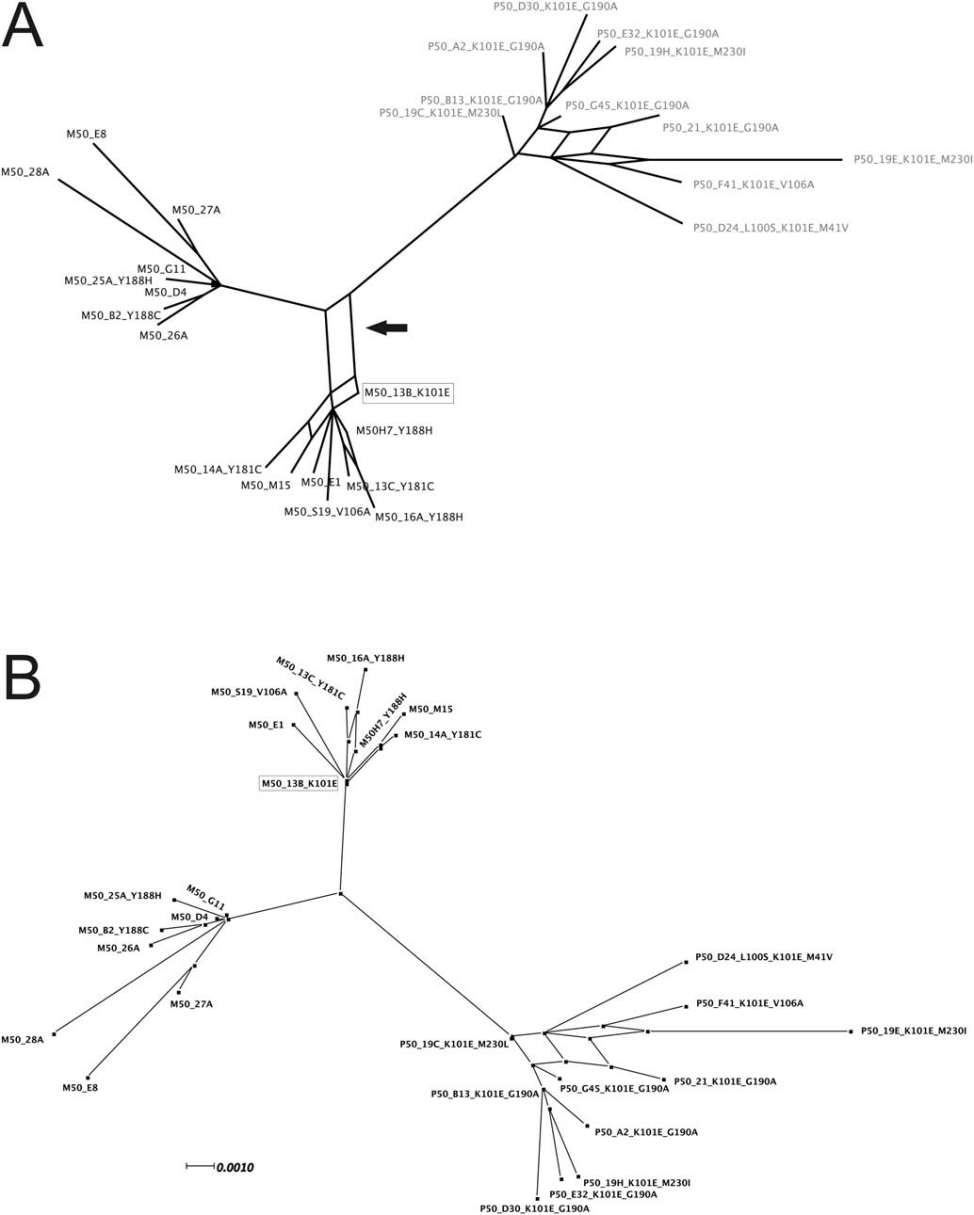
NeighborNet phylogenetic analysis of M50 and P50 viral sequences including (*A*) or excluding (*B*) the RT codon 101. The formation of a net (pointed by the arrow) in *A* depicts genetic relatedness between sequence M50_13B_K101E and the child viral sequence cluster. M50_13B_K101E is boxed in both panels.

Phylogenetic analysis of M50-P50 clonal sequences and additional controls clearly showed the mother-to-child transmission linkage with high bootstrap support. Only sequences larger than 780 bp were included for clarity of phylogenetic signal (Figure 1). A well-supported cluster was inferred for both the transmission chain and the individual patient sequences. The phylogenetic relationships between the mother and the child clones are presented in more detail in Figure 2. The K101E-containing variant from the mother (M50_13B_K101E) showed phylogenetic evidence of common ancestry with the child cluster, as seen by the net formed by both clusters (Figure 2A). Although exclusion of codon 101 from the analysis failed to show such linkage, M50_13B_K101E still remained at the base of the mother cluster, as one of the sequences from the mother most similar to the child cluster (Figure 2B).

**Figure 3:**
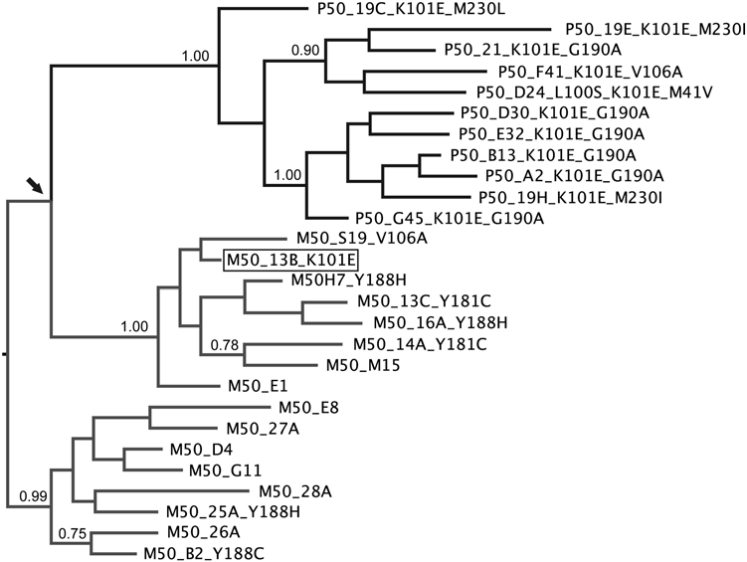
Majority-rule consensus tree of the Bayesian MCMC analyses. Only posterior probablities >0.75 are shown. The maximum likelihood tree showed a very similar clustering. The arrow indicates the closer relatedness between the mother sequence cluster harboring M50_13B_K101E (boxed) and the child cluster.

The Bayesian MCMC analysis revealed that the cluster from the mother harboring sequence M50_13B_K101E was indeed more closely-related to the child cluster (Figure 3). Taken together, all phylogenetic evidences suggest that M50_13B_K101E, the only viral sequence from the mother carrying K101E, share common ancestry with viruses circulating in P50.

## Discussion

We have reported here the description of MTCT of a minor HIV-1 variant carrying the NNRTI mutation K101E. The child HIV variant evolved shortly after treatment with 2 NRTIs to a predominant species also carrying the G190A mutation, which confers resistance to all NNRTI, albeit in the absence of NNRTI selective pressure.

K101E mutation can cause resistance to nevirapine and a low level resistance to efavirenz and etravirine [15]–[16]. The fitness of variants containing solely the K101E mutation remains to be determined but its persistence in the child along all her follow-up and our phylogenetic evidences suggest that this variant was the founder species transmitted to the child even though it was present in less than 0.3% of the mother's viral quasispecies.

Our standard genotyping showed that K101E was already present in the first sample of the child (before treatment) but only the minority of the virus population had the NNRTI major mutation G190A. The standard genotyping was able to detect G190A mutation only one month after start of ARV, when the T215F mutation was already present (Table 1). The fact that G190A was not observed in the mother's genotyping may suggest that it could have emerged *de novo* in the backbone containing K101E during evolution of the child quasispecies. G190A is known to impact only slightly in viral fitness [17], and could have remained in the quasispecies until selected as a major variant by the emergence of T215F and NRTI selective pressure. Alternatively, it may have faded away over time from the mother's virus population, as her first sample was collected 4 years after her HIV diagnosis. The mother presented some minor HIV variants carrying NNRTI resistant mutations, suggesting that she was infected with a NNRTI primary resistant virus. Finally, G190A could be present in a very small frequency in the mother's viral quasispecies, below the detection limit of the assays. Wang *et al.* [18] have shown that certain NRTI mutations, including T215Y, can increase the fitness of K101E+G190S variants. We speculate that T215F has emerged in those viral species already carrying both NNRTI mutations, and the selective pressure of NRTI in the child propitiated the emergence of G190A-containing strains as major variants.

Although the horizontal transmission of HIV minority resistant strains [19] and the vertical transmission of resistant strains [20] have been both previously reported, this is the first documentation, to the best of our knowledge, of MTCT of minority resistant strains of HIV-1.

Our report highlights the importance of transmission of resistant minority strains, as it carries the threat of emergence of multi-drug resistance primary mutations without specific selective pressures. Pediatricians who provide care to HIV-infected children should be aware of this possibility, especially when dealing with children who acquired HIV by vertical transmission and whose mothers were treated with ARV during pregnancy. The use of ultrasensitive genotyping technologies for the detection of drug resistance in MTCT cases may not be feasible for most developing countries. In this setting, the early use of standard genotyping tests in children with virologic failure during treatment can promote the detection of unsuspected resistant mutations and optimization of their future ARV regimens.
